# The Prevalence of unexpected antibodies in Saudi's plasma prior blood transfusion and their association with clinical conditions: A cross-sectional study

**DOI:** 10.1016/j.sjbs.2021.04.083

**Published:** 2021-05-07

**Authors:** Hisham Ali Waggiallah, Faris Q. Alenzi, Abdulkarim S. Bin Shaya, Ahmed Hattan Hattan, Yousif Mohammed Elmosaad, Maher M. Alenazi

**Affiliations:** a Department of Medical Laboratory Sciences, College of Applied Medical Sciences, Prince Sattam Bin Abdulaziz University, Saudi Arabia; b Department of Laboratory, King Fahad Medical City, Saudi Arabia; c Department of Public Health, College of Applied Medical Sciences, King Faisal University, Saudi Arabia

**Keywords:** Unexpected antibody, Blood group, Blood transfusion

## Abstract

Unexpected antibodies, also called irregular antibodies, are not known to exist in a person's serum before testing. This research aims to assess the prevalence of unexpected antibodies and their correlation with several clinical conditions. This cross-sectional prospective study, undertaken from June 2019 to June 2020, included ABO, Rh grouping, cross-matching, and antibody screening. Antibody identification was performed only on patients who tested positive in the screening test. From a total of 9764 participants who were screened for unexpected antibodies, 107 (1.1%) tested positive. The Rh blood group system antibodies were the most frequent, particularly anti-D. There was also a significant correlation between the unexpected antibodies and history of transfusion, pregnancy, and autoimmune diseases as P ≤ 0.05. The most prominent unexpected antibodies in the study belong to the Rh system (Anti-D). Moreover, as a result of the strong correlation between the unexpected antibodies as well as the history of transfusion, pregnancy, and autoimmune diseases, the highest safety criteria must be followed during the transfusion of blood to patients with these clinical conditions.

## 1 Introduction

Blood compatibility testing is necessary to prevent critical complications during blood transfusion. The American Association of Blood Banks (AABB) has recommended that ABO typing, Rh typing, unexpected antibody screening, and cross-matching must be performed before a blood transfusion. Since blood antibody screening is generally included in the list of preoperative tests, an emergency transfusion can be performed immediately in the case of massive intraoperative bleeding. However, when an emergency transfusion is required in circumstances in which the unexpected antibody screening test has not been performed, the delay in finding compatible blood may be life-threatening (Kim et al., 2013).

Unexpected antibodies, also called irregular antibodies, cannot be confirmed to exist in a person's serum until it is tested. They include antibodies and antigens from systems such as Rh, MNSs, Duffy, and Kidd (Mazonson et al., 2014). Unexpected antibodies have quite a major impact since they can cause serious transfusion reactions such as acute or delayed hemolytic transfusion reaction or neonate hemolytic syndrome. Blood grouping and cross-matching is one of the most important tests to conduct before blood transfusion. Therefore, proper knowledge of the blood group system, its clinical significance, typing and cross-matching procedures, and experience is vital in the prevention of transfusion-related complications (Getshen et al., 2019). Furthermore, knowledge of the blood group system is required to tackle diseases linked to blood group (Mazonson et al., 2014).

RBC alloantibody formation is uncertain in patients who have previously been transfused or pregnant. Further, the alloimmunization rate has been documented to be 1–10% and even higher (20%) in regularly transfused populations (sickle cell disease or thalassemia patients). Pre-transfusion testing, particularly antibody screening, has been developed to identify these antibodies, and antibody detection (ABI) techniques are designed to detect different antibodies (Goss et al., 2016).

This research aims to determine the occurrence of unexpected antibodies in Saudi patients prior to blood transfusion and to study their association with several clinical conditions.

## 2 Materials and methods

This prospective cross-sectional study was conducted among hospitalized patients needing blood transfusions from June 2019 to June 2020.

### 2.1 Inclusion criteria

Regardless of sex and age, only Saudi patients who were hospitalized for blood transfusion were included in this study.

### 2.2 Exclusion criteria

Patients who have a history of hematological malignancies were excluded.

A total of 9764 blood samples were obtained. The designed questionnaire was used for the collection of demographic and clinical data by a well-trained data collection team. All of the following techniques were verified by hematology and blood banking experts according to the standard procedures operation manual.

### 2.3 ABO and Rh grouping

ABO and Rh typing should be done for the blood of both the donor and the recipient. ABO typing is performed by testing RBCs with anti-A and anti-B antisera and A1 and B serum. Rh type is determined with the anti-Rh, anti-D; if the donor’s initial anti-D blood test result is negative, then the blood is screened using a weak D (Du) detection method. If an Rh test result is positive, the blood sample’s label will read “Rh positive” (AABB, 1993) (Bio-Rad Laboratories, Inc.).

### 2.4 Antibody screening test

The patient’s serum or plasma should be screened for a single-donor suspension of unpooled RBC group O reagent. This is in compliance with AABB Standards (AABB, 1993). Such reagent cells are chosen because they bear the blood group antigens required for the identification of the most “clinically important” RBC alloantibodies, i.e., those antibodies reactive at 37 °C in the AHG process of cross-matching as compared to those reactive at room temperature. A few drops of serum and one drop of reagent cells are incubated at 37 °C for 30 min, centrifuged, checked for hemolysis and agglutination, washed three to four times with normal saline, and checked with anti-IgG using the indirect antiglobulin test (IAT). Once received, test findings are first read macroscopically for agglutination and then microscopically if the result is negative (AABB, 1993) (Bio-Rad Laboratories, Inc.).

### 2.5 Cross-matching test

The patient’s serum screening with red cells from the donor should be conducted prior to the transfusion of the whole blood or red cell components. For patients who have been tested negative for red cell alloantibodies and who have no history of these antibodies, only an instant spin crossmatch (IS-XM) can be done to test ABO compatibility. Moreover, cross-matching is performed by combining two to four drops of the patient’s serum with one drop of the donor’s RBCs suspended in a 2–5% saline solution in a test tube, spinning it instantly, and then checking for hemolysis and agglutination. In such situations, the AHG crossmatch is not necessary. Contrarily, the AHG crossmatch (AHG-XM) should be done in patients who test positive for RBC alloantibodies or have a history of clinically significant antibodies. In AHG-XM, IS-XM is proceeded by incubation at 37 °C for 30 min, followed by anti-IgG IAT. If the patient critically requires blood transfusion and there is not enough time to undertake a routine study, a shorter incubation period (10 min) can be observed using a low-ionic additive solution (Anderson Cancer Center, 1993) (Bio-Rad Laboratories, Inc.).

### 2.6 Antibody identification

If the antibody screening detects one or more antibodies in the patient’s plasma, further testing should be conducted to identify them. This procedure, defined as antibody identification, involves checking the patient's plasma with a red blood cell type O panel with known antigen expression. Antibody detection requires several steps designed to eliminate and validate particular antibodies, assess the temperature for optimum antibody reactivity, and classify any autologous reactivity. Antigen typing of the patient’s red blood cells can also help in the detection of antibodies by recognizing which RBC antibodies and individuals are prone to creating them. Finally, the time needed to complete an antibody identification is extremely variable as it depends on the size of the antibody investigation required (Shulman et al., 1990) (Bio-Rad Laboratories, Inc.).

### 2.7 Statistical analysis

The collected data was analyzed using the statistical package for the Social Sciences (SPSS) version 22. In addition, a frequency test was used for the distribution of demographic data, clinical conditions, and unexpected antibodies in various blood group systems. Lastly, a chi-square test was used to reveal the correlation between unexpected antibodies and autoimmune diseases, history of blood transfusion, and pregnancy.

### 2.8 Ethi cal approval

The ethical clearance for the research was obtained from the institutional review board at Prince Sattam Bin Abdulaziz University. Written consent was obtained from the participants after a productive discussion. They were informed that the result of the study would be utilized for their benefits.

## 3 Results

Of the 9764 patients tested for unexpected antibodies, 107 (1.1%) tested positive as shown in Table 2. The distribution of the demographic data revealed that the age group of 21–40 years had the most frequent unexpected antibodies (45%) while female participants had a higher number (77%) than the males. Regarding the prevalence of unexpected antibodies among the ABO blood group system, it was found that the O blood group had a higher percentage (45.8%) although the positive Rh system also comprised a high percentage (64.5%).

Most of the participants have a history of blood transfusion (69.2%) or pregnancy (53.3%). Participants with autoimmune diseases constituted 78.5% whilst those with immune hemolytic anemia constituted 40.9% as the highest percentage (Table 1 ). The researchers found that the Rh system has the most frequent unexpected antibodies (65.3%), especially anti-D (29%) and anti-E (18.7%) and, to some extent, the Kidd blood group system with anti-JKa (11.2%) as exhibited in Table 2. The correlation between unexpected antibodies and patients with autoimmune diseases, history of blood transfusion, and pregnancy is significant since the p-value is less than or equal to 0.05 (Table 3).

**Table 1:** Shows frequency of demographic and clinical data among Saudi population.

Parameters	Frequency	Percentage
**Age group in years**		
● 1–20	26	24.3
● 21–40	45	42.1
● 41–60	24	22.4
● 61–80	12	11.2
**Gender**		
● Male	28	30
● Female	72	77
**ABO blood group system**		
● A	30	28.0
● B	21	19.6
● O	49	45.8
● AB	7	6.5
**Rh blood group Group**		
● +ve	69	64.5
● −ve	38	35.5
**History of transfusion**		
● None	33	30.8
● Once	24	22.4
● Twice	18	16.8
● Three times	15	14.0
● Four times	17	15.9
**History of pregnancy**		
● None	50	46.7
● Once	15	14.0
● Twice	15	14.0
● Three times	7	6.5
● Four times	8	7.5
● Five times	12	11.2
**Auto immune disease**		
● None	23	
● ITP	20	18.7
● SLE	15	14.0
● RA	6	5.6
● AIHA	43	40.9

ITP: Idiopathic thrombocytopenia, SLE: Systemic lupus erythematosus, RA: Rheumatoid arthritis, AIHA: Autoimmune hemolytic anemia.

**Table 2:** Frequency of unexpected antibodies among selected Saudi population.

Parameters	Frequency	Percentage
Negative of screening test	9657	98.9
Positive screening test	107	1.1
**Unexpected antibodies details (n = 107)%**		
**Parameters**	**Frequency**	**Percentage**
**Rh system**		
Anti D	31	29
Anti C	10	9.3
Anti E	20	18.7
Anti c	1	0.9
Anti E + C	1	0.9
Anti D + C	7	6.5
**Kidd system**	–	–
Anti JK^a^	12	11.2
**Kell system**		
Anti K	8	7.5
**MNSs system**	–	–
Anti M	2	1.9
Anti S	5	4.7
**Lewis system**		
Anti Le(a)	6	5.6
**Mixed antibodies**	–	–
Anti M + Anti E	1	0.9
Anti S + Anti E	1	0.9
Anti K + *Anti Fy^a^	2	1.8

*Anti Fy^a^ belong to Duffy blood group system.

**Table 3:** Illustrated correlations between unexpected antibodies, autoimmune diseases, history of blood transfusion and history of pregnancy.

Parameters			Frequency (%)	X^2^	P-value
Unexpected antibodies	Autoimmune Diseases	With autoimmune diseases	84 (78.5%)	24.6	0.026*
		Without autoimmune diseases	23 (21.5%)		
	History of Blood Transfusion	No history of Transfusion	33 (30.8%)	20.7	0.024*
		1–2 times transfused	42 (39.3%)		
		3 times transfused	32 (29.9%)		
	History of Pregnancy	No history of pregnancy	34 (47.2%)	18.7	0.045*
		1–2 times pregnancy	25 (34.7%)		
		More than 3 times	13 (18.1%)		

*P ≤ 0.05 is significant.

## 4 Discussion

In the research, the ratio of unexpected antibody prevalence (1.1%) is on par with previous studies; Ho Ko et al. (2012) recorded the prevalence of unexpected antibodies to be 1.35% in Denmark, 0.78% in Germany, and 0.3–2% in the United States (Ko et al., 2012). This ensures that blood transfusion criteria and policies were perfectly implemented in the blood banking sector as per global standards. Blood safety relies on the recruiting and retention of blood donors who are at lower risk of transmitting infection; healthy blood collection procedures; appropriate testing for transfusion-transmissible infections; blood grouping and compatibility screening; and acceptable and healthy use of blood (Blood Transfusion Safety, 2006).

According to the demographic data, females constituted the vast majority of participants as they were desperate for blood transfusion because of pregnancy, abortion, cesarean surgery, and heavy menstrual cycles.

It is commonly understood that the significant variations present in the occurrence and formation of unexpected antibodies are identified while screening by testing blood type, genotypic incidence, screening process, and decoder capability. When positive antibody screening values are produced prior to blood transfusion, an antibody detection test is performed to assess the nature of the antibody. If its nature is known, the proportion of the provided blood that tested negative for the pertinent antibody is determined (Cheng et al., 2012). Further, alloimmunization reactions to the RBC’s antigens arise as unexpected antibodies are produced following the introduction to RBC antigens that are unfamiliar to the patient. These could happen as a result of transfusion or pregnancy. Consequently, antibody development could occur from an anamnestic reaction, since the same RBC antigen is repeatedly exposed (Hart et al., 2015). Antibodies do not develop weeks or even months following their preliminary reaction to foreign RBC antigens. According to the prolonged exposure to the antigens, antibodies may be produced quickly 48–72 h after transfusion and reach the highest titer 7–10 days later. Between the modes of alloimmunization reactions, hemolytic reactions to the recently produced antibody are categorized as anamnestic hemolytic reactions, whereas reactions with the absence of hemolysis are categorized as delayed serolytic reactions (Bahri et al., 2018).

The findings of this study, in which Rh anti-D is identified as the most frequent antibody, followed by anti-E, are inconsistent with the previous study conducted in Saudi Arabia, which reported that the most frequent Rh system antigen is anti-D, observed in about 80% of the population (Owaidah et al., 2020). However, our research agrees with that of Schonewille et al. (2006), conducted in Netherlands, and with that of Chen et al. (2016), performed in China, which found that among all the reported antibodies, those of the Rh blood group system were the most frequent, followed by the MNS, Lewis, Kidd, Duffy, Diego, and Kell systems. However, our research did not agree with Höglund et al., 2013, Han et al., 1989, who reported that the Lewis blood group anti-Le(a), which is believed to trigger anamnestic hemolytic reactions, was the most frequently detected antibody,. The facts that anti-Le(a) antibodies are often produced not by immune reactions but naturally and that the patient in their case had no history of pregnancy or transfusion were cited as the reasons for their findings. This is emphasized by our research; most of the participants have a history of blood transfusion and pregnancy and, at the same time, have low frequency anti-Le(a). Another study conducted in India by Sahoo et al. (2020), found that the red cell alloimmunization among Rh-D negative women and Rh-D positive pregnant women was 4.42% and 2.2% respectively. The antibodies identified in this study were anti-D, anti-C, anti-E, anti-C, and anti-Le(a) (Sahoo et al., 2020).

Anti-JKa and Anti-K are a major alloantibody that can induce HDN or severe HTRs (Sahoo et al., 2020). The production of such an antibody is primarily due to transfusion or pregnancy; the explanation for this finding is that, given the specificity of RBC antigen immunogenicity, the antigen presence rate may differ significantly depending on the ethnicity group. Since HDN-caused JKa antibodies are uncommon, physicians should be conscious that the incidence of such antibodies may progress to extreme HDN and chronic anemia in infants (Mittal et al., 2016).

Anti-M, anti-S, and anti-Fya antibodies were the least frequent unexpected antibodies in this research alongside mixed antibodies. Unexpected antibodies that occur in small concentrations cannot be identified by blood compatibility tests, but can lead to unexpected hemolytic reactions due to the transfusion of RBCs that respond to antibodies. In fact, antibody concentrations may be escalated following repeated immune reactions, which can lead to severe hemolytic transfusion reactions during the next transfusion. To minimize the occurrence of delayed hemolytic reactions, the serum must be obtained and checked every 72 h for patients requiring numerous transfusions, or an extremely specific screening procedure must be utilized for the compatibility test (Sandeep and Anupam, 2014).

The participants in the research showed a strong correlation when comparing autoimmune diseases, history of blood transfusion, and pregnancy with the presence of unexpected antibodies (P ≤ 0.05). This means that the fundamental causes of the production of unexpected antibodies are due to the factors mentioned above. Therefore, the decision to recommend transfusion to patients with autoimmune hemolytic anemia should be focused on the patient's reaction to hemolysis, the cause of immunohemolytic anemia, and the form of antibodies that contribute to this disorder. It is improbable that patients without any history of pregnancy or transfusion will have alloantibodies beside autoantibodies; therefore, appropriate transfusion of blood could be obtained without an autoantibody absorption test (ZZAP test). Further, the presence of alloantibodies must be taken into consideration while choosing compatible blood for patients receiving numerous transfusions or for women who have a history of frequent delivery (Kathpalia et al., 2016, Shin, 2013).

Autoantibodies are difficult to distinguish from alloantibodies because both react in similar manner with RBCs. Therefore, an attempt must be made to identify compatible blood if alloantibodies and autoantibodies are present. In contrast, there are fewer requirements to identify compatible blood in a case where only autoantibodies exist. Thus, for patients who only have autoantibodies, most blood banks transfuse RBCs that display the lowest degree of reaction in cross-matching. In patients with auto and alloantibodies, RBCs that test negative for specific antigens that give the smallest reaction are transfused. It may be difficult to identify compatible blood quickly; therefore, the anesthesiologist should identify the existence of unexpected antibodies prior to surgery, ensuring timely and safe transfusion by close collaboration with the surgeon and the blood bank (Kim et al., 2013).

In patients who test positive for unexpected antibodies, blood that is negative for antigens may induce a delayed reaction and must be screened and transfused. Many medical services provide patients with medical warning cards containing these details while they are admitted in hospital and receiving transfusion (Sachan et al., 2015).

Fortunately, the production of antibodies due to anamnestic reactions usually causes only delayed serological reactions. Furthermore, when there is an urgent necessity for transfusion during an operation, the physician should be aware of the likelihood of emergencies and the risk of transfusion delays. This is especially relevant for patients with unexpected antibodies for whom it may be difficult to find compatible blood for transfusion (Ho Ko et al., 2012, Brecher, 2002).

## 5 Conclusion

A strong correlation has been found between the emergence of unexpected antibodies, especially Rh system (anti-D), with patients’ immune history, and it is a reasonable correlation in comparison with global ratios. Precautions should be applied as the development of these antibodies may lead to autoimmune hemolytic anemia, which endangers the lives of patients. Therefore, following the protocols used for blood transfusion necessarily reduces the effects of these antibodies.

## Funding

This study was supported by Deanship of Scientific research at Prince Sattam Bin Abdul aziz University under the research project No 2019/03/16546.

## Declaration of Competing Interest

The authors declare that they have no known competing financial interests or personal relationships that could have appeared to influence the work reported in this paper.
